# The importance of digitalisation and data science for HIS strengthening

**DOI:** 10.1093/eurpub/ckac129.134

**Published:** 2022-10-25

**Authors:** D Novillo

**Affiliations:** Data and Digital Health Unit, WHO Regional Office for Europe, Copenhagen, Denmark

## Abstract

The COVID-19 (coronavirus disease 2019) pandemic has underscored the critical need for all countries to strengthen their health data and information systems and ensure the routes the data travel, from submission to use, are unobstructed. Timely, credible, reliable, and actionable data are key to ensuring that political decisions are data driven and facilitate understanding, monitoring, and forecasting. To ensure that critical decisions related to the wider health and socioeconomic effects of this pandemic are data driven, each country needs to develop or enhance a national data governance plan that includes a clear coordination mechanism, well-defined and documented data processes (manual or electronic), the exchange of data, and a data culture to empower users. In addition, countries should now more than ever invest and enhance their data and health information systems to ensure that all decisions are data driven and that they are prepared for what is next. Furthermore, strong enabling environments and advanced and digitized health information systems are vital to controlling epidemics. Sustainable finance and government support are required for the continued implementation and enhancement of HIS. It is important to promote digital solutions beyond the COVID-19 pandemic. Now is the time to discuss potential solutions to obtain timely, accurate, and reliable health information and steer policy-making while protecting privacy rights and meeting the highest ethical standards.

